# Transforming Basic Literacy Instruction through the Kapal Pinisi Application: Integrating Local Wisdom to Foster Basic Literacy and 6C Competencies in Elementary School Students

**DOI:** 10.12688/f1000research.172137.1

**Published:** 2026-03-04

**Authors:** Gunawan, Alfi Laila

**Affiliations:** 1 Indonesian Language Education Study Programme, Faculty of Teacher Training and Educ, Universitas Muhmmadiyah Palembang, Palembang, Sumatera Selatan (South Sumatra), 30263, Indonesia; 2 Faculty of Teacher Training and Education, Universitas Nusantara PGRI Kediri, Kediri, East Java, 64112, Indonesia

**Keywords:** Kapal Pinisi application, local wisdom, basic literacy, 6C competencies, elementary education, digital learning innovation, literacy transformation

## Abstract

**Background:**

The persistent lack of basic literacy and 6C competencies—Critical Thinking, Creativity, Communication, Collaboration, Citizenship, and Character—among elementary school students poses a significant challenge to achieving equitable educational quality. Traditional literacy programs often neglect the integration of cultural values and technological innovation. To address this gap, this study developed the *Kapal Pinisi Application*, a digital learning medium rooted in Indonesian local wisdom, designed to strengthen students’ basic literacy and 21st-century competencies.

**Methods:**

This research adopted a Research and Development (R&D) approach using the ADDIE model, encompassing the stages of analysis, design, development, implementation, and evaluation. The application was implemented among 200 elementary students from ten schools across Indonesia. Data were collected through pretest and posttest assessments during small- and large-scale trials. The N-Gain test was employed to analyze improvement in learning outcomes, and ethical clearance was obtained from the Research Ethics Committee of Universitas Muhammadiyah Surakarta.

**Results:**

Findings revealed a significant improvement in students’ basic literacy and 6C competencies after using the *Kapal Pinisi Application.* The N-Gain analysis yielded an average score of 0.88, categorized as high effectiveness. Notable progress was observed in reading comprehension, numeracy, creativity, collaboration, and character development. The integration of cultural elements enhanced student engagement, contextual understanding, and motivation.

**Conclusions:**

The *Kapal Pinisi Application*, which harmonizes local wisdom and digital innovation, proved highly effective in improving basic literacy and 6C competencies among elementary school students. This model demonstrates the potential of culturally grounded, technology-enhanced learning media to promote literacy transformation aligned with 21st-century education and sustainable development goals.

## Introduction

Many elementary school students in Indonesia still encounter difficulties in mastering basic literacy, namely reading, writing, and arithmetic, and have not yet adequately developed the 6C skills (Communication, Collaboration, Critical Thinking, Creativity, Citizenship, and Character). This challenge is even more pronounced in schools located in rural and semi-rural areas with limited access to educational resources. In contrast, the demands of the 21st century emphasize that students are expected not only to master academic content but also to acquire life skills and character that enable them to adapt to the digital era, global society, and rapid social transformations. Although literacy learning initiatives have been implemented in schools, their results remain suboptimal. A study ^1^ conducted in Boyolali, a semi-rural district in Central Java Indonesia, shows that although schools in the area have planned and organized literacy programs, more intensive efforts in teaching, habituation, and evaluation are still required to ensure equitable student literacy outcomes. Another study ^2^ highlights a significant correlation between the quality of textbooks and the basic literacy skills of lower elementary school students, reinforcing the importance of instructional resources. Furthermore, a preliminary study ^3^ revealed that there is an urgent need for innovative learning media that integrate local wisdom and support the development of students’ 6C skills. The study emphasizes that conventional literacy programs and existing digital tools are insufficient if they do not combine literacy with contextual cultural values, interactive features, and collaborative learning approaches. These findings suggest that conventional literacy programs alone are insufficient. Therefore, innovative learning media are needed not only to strengthen basic literacy but also to simultaneously foster the comprehensive development of the 6C competencies in an integrated manner. For instance, a systematic literature review of e-learning platforms shows that digital learning media, multimedia, and learning management systems significantly improve literacy and critical thinking skills among students in vocational contexts. ^4^ Blended learning models have also been identified to have positive impacts on student engagement, autonomy, academic outcomes, and 21st-century skills, including communication and collaboration. ^5^

Recent educational literature supports the idea that integrating local cultural literacy with digital learning models is highly beneficial in strengthening basic literacy and 21st-century competencies. ^6, 7^ This integration refers to the combination of local wisdom-based content (such as traditional stories, values, and cultural practices) with technology-enhanced instructional media that promote active and responsive learning. However, there is still little empirical testing of such digital applications that embed local cultural elements in a broad elementary school context. Another study found that education emphasizing character development, utilizing ethnopedagogical approaches and local wisdom, significantly strengthens students' character and citizenship dimensions. ^8^ Based on this, it indicates that there is a strong theoretical and empirical foundation for developing media or applications that combine basic literacy, technology, and local elements; however, specific research such as the Kapal Pinisi application in the context of elementary schools and the comprehensive measurement of all 6Cs is still limited. ^8^

The main objective of this paper is to evaluate the effectiveness of the Kapal Pinisi application, which is based on local wisdom, in improving basic literacy and the 6C skills of elementary school students in Indonesia. Because if the application proves effective, it can serve as a scalable, culturally relevant, and sustainable model for learning media that addresses urgent basic literacy needs while fostering comprehensive 21st-century competencies. Therefore, this research aims not only to measure the improvement of basic literacy through the application, but also whether the application is empirically and significantly able to develop the six 6C competencies, thus contributing to educational practices and policies in Indonesia.

The use of the Kapal Pinisi application, which integrates technology and local wisdom, is expected to contribute to the improvement of elementary school students’ basic literacy and 6C competencies compared to conditions prior to the intervention. This expectation is grounded in theoretical and empirical evidence suggesting that contextual, culturally relevant, and interactive learning media that combine literacy reinforcement with soft skills and character development may result in stronger improvements in literacy mastery and 21st-century competencies than traditional approaches without local integration. ^6, 9^ It is therefore anticipated that the Kapal Pinisi application could serve as an effective intervention and has the potential to be recommended as part of a broader national strategy to strengthen basic literacy and foster students’ 6Cs in elementary schools. In this context, local wisdom and citizenship-based multiliteracy have emerged as strategic approaches for addressing the challenges of 21st-century education. Multiliteracy is no longer understood solely as traditional reading and writing skills, but rather as the ability to access, interpret, and generate meaning across diverse forms of representation, including textual, visual, digital, and symbolic modes. ^10, 11^ Social change and technological advancements require students to acquire contextual multiliteracy skills that enable them to adapt to real-life demands and the dynamic world of work. Accordingly, multiliteracy provides opportunities for the development of critical and reflective thinking in response to increasingly complex information. Thus, the multiliteracy approach is considered essential in primary education, as it connects conventional literacy skills with 21st century competencies and offers a solid foundation for preparing younger generations to face rapid and diverse global transformations.

The application of multiliteracy in primary education is highly relevant for developing 21st-century competencies, which include critical thinking skills, creativity, communication, collaboration, digital literacy, and character building. ^12, 13^ Integrating local wisdom into multiliteracy plays an important role in ensuring that learning is not uprooted from students’ cultural roots, while also providing contextual meaning to the learning experience. Local wisdom serves as a bridge connecting traditional values with global demands. ^14, 15^ Cultural values such as mutual cooperation, deliberation, and tolerance, when integrated into multiliteracy learning, have been proven to strengthen civic literacy and build students’ character. ^16^ Thus, multiliteracy based on local wisdom not only develops academic skills but also supports the formation of cultural and moral identities that align with the demands of the digital age.

The dimension of digital literacy within multiliteracy is also an urgent need in the information technology era. Students are not only expected to be able to access information, but also to use it critically, ethically, and productively in their daily lives. ^17, 18^ Nevertheless, there are significant challenges that still need to be addressed, including the gender gap and limited access to technology, which lead to inequalities in digital literacy. ^19^ Thus, integrating local wisdom into multiliteracy can serve as a strategy to balance technological modernization with cultural values. This approach not only preserves local identity but also helps mitigate the negative impacts of digitalization, such as cultural homogenization and excessive dependence on technology. Therefore, strengthening multiliteracy based on local wisdom is a relevant solution to harmoniously blend traditional values and digital skills.

Additionally, multiliteracy that integrates citizenship dimensions provides an important space for students to develop as critical, democratic, and character-driven citizens. Civic literacy focuses on social participation skills and collective responsibility, which are currently becoming increasingly crucial amidst global dynamics. ^20^ showed that local culture-based citizenship education is able to increase civic engagement and strengthen social cohesion, which ^21^ findings emphasizing the role of education in building social solidarity. Thus, multiliteracy based on local wisdom and citizenship is not merely a pedagogical model, but a transformative strategy to prepare students to face global challenges without losing their cultural identity. This theoretical foundation asserts that the combination of multiliteracy, local wisdom, and civic literacy is an integrative approach to building 6C competencies while simultaneously strengthening national character.

## Methods

This research employed a Research and Development (R&D) approach by adopting the ADDIE model, which consists of five main stages: (1) analysis, (2) design, (3) development, (4) implementation, and (5) evaluation. The effectiveness of the **Kapal Pinisi** application was tested in two stages: small-scale and large-scale trials. ^22^ Data were collected through pretest and posttest, and analyzed using the N-Gain test. Ethical approval was obtained from the Research Ethics Committee of Universitas Muhammadiyah Surakarta.

### Sampling strategy and research context

The study involved 200 elementary school students from ten schools in Indonesia, with data collection conducted in October 2024. A purposive sampling technique was used to select the schools and students. The criteria included: (a) representation from both public and private institutions, (b) schools situated in both urban and semi-rural settings, (c) diversity in resource availability, and (d) the willingness of school principals and teachers to collaborate in the research. The small-scale trial involved 50 students at SDN Ngampel 2 and SDN Bandarlor 3, while the large-scale trial included 150 students from eight other schools, namely SDN Besuk 1, SDN Mlancu 2, SDN 22 Lanjau, SDN 1 Sumberbaru, SDN Bagelenan 3, SDN 15 Merawang, SDN 95 Gresik, and SD Kristen Abat. These schools represent a combination of urban and semi-rural contexts with varied cultural backgrounds, making them suitable for testing the integration of local wisdom into digital literacy learning.

### Integration of the Kapal Pinisi application into classroom practice

The **Kapal Pinisi** application was implemented as a supplementary digital learning medium integrated into existing literacy lessons. Its use was teacher-facilitated: teachers introduced and guided students in operating the application during scheduled learning sessions. Students then interacted with the application either individually or in small groups. The application did not replace the existing curriculum; rather, it complemented classroom instruction by providing culturally relevant, interactive content designed to reinforce students’ basic literacy skills while simultaneously nurturing their 6C competencies.

### Key features of the Kapal Pinisi app

**Local culture-based basic literacy content**

1. Reading materials are integrated with Indonesian cultural stories.
2. Students learn literacy while gaining knowledge of customs, traditional dances, clothing, and local beliefs.
3. This feature helps address the issue of literacy being perceived as irrelevant to real-life contexts.

**Nusantara cultural exploration**

1. **Traditional Foods:** For example, *rendang*, *papeda*, and *sate lilit* presented with virtual interaction.
2. **Traditional Games:** Such as *congklak*, spinning tops, and marbles—offered not only as descriptions but also as virtual simulations.
3. This responds to the lack of students’ knowledge of local wisdom, which is increasingly overshadowed by foreign digital media.

**6C-based games**

1. Interactive challenges designed to train *Critical Thinking, Creativity, Collaboration, Communication, Citizenship,* and *Character.*
2. Example: a collaborative game to create cultural stories, which fosters teamwork and creativity.
3. Addresses the gap in literacy learning that often emphasizes cognitive aspects while neglecting 21st-century skills.

**Interactive quizzes**

1. Customized for students, teachers, and the community.
2. Formats include multiple-choice, matching, and true/false items.
3. Provides rapid assessment of basic literacy and cultural understanding.
4. Responds to the issue of insufficient evaluation in literacy initiatives.

**Child-friendly design (User-friendly UI/UX)**

1. Animated 2D interface with student and teacher cartoon characters.
2. Includes an illustrated map of Indonesia and the Kapal Pinisi icon, promoting a sense of belonging.
3. Helps overcome the lack of engagement often associated with conventional learning media.

**User segmentation: Students – Teachers – Community**

1. **Students:** Focus on basic literacy, culture, and traditional games.
2. **Teachers:** Access to interactive teaching resources (financial literacy, parenting, cultural literacy).
3. **Community:** Literacy related to disaster mitigation, economy, and agriculture.
4. Addresses the issue of literacy being seen solely as the responsibility of schools rather than a collective effort.

**Highlighted features relevant to critical issues**

1. **Integration of literacy, culture, and technology** → A solution to the disconnect between school literacy programs and local contexts.
2. **6C-based games** → Addressing the limited development of 21st-century competencies in elementary schools.
3. **Multi-user segmentation (students, teachers, community)** → Strengthening the literacy support system.
**Suggested illustrations**

Kapal Pinisi App

├── Basic Literacy (Cultural stories)

├── Cultural Exploration (Foods, games)

├── 6C Games (Critical Thinking, Creativity, etc.)

├── Interactive Quizzes

└── Segmentation (Students – Teachers – Community)

**UI screenshot/Mockup**

1. Selection page of “Islands” for students, teachers, or community users.
2. Illustration of a virtual *congklak* game or the process of cooking *rendang.*

**Figure 1.**
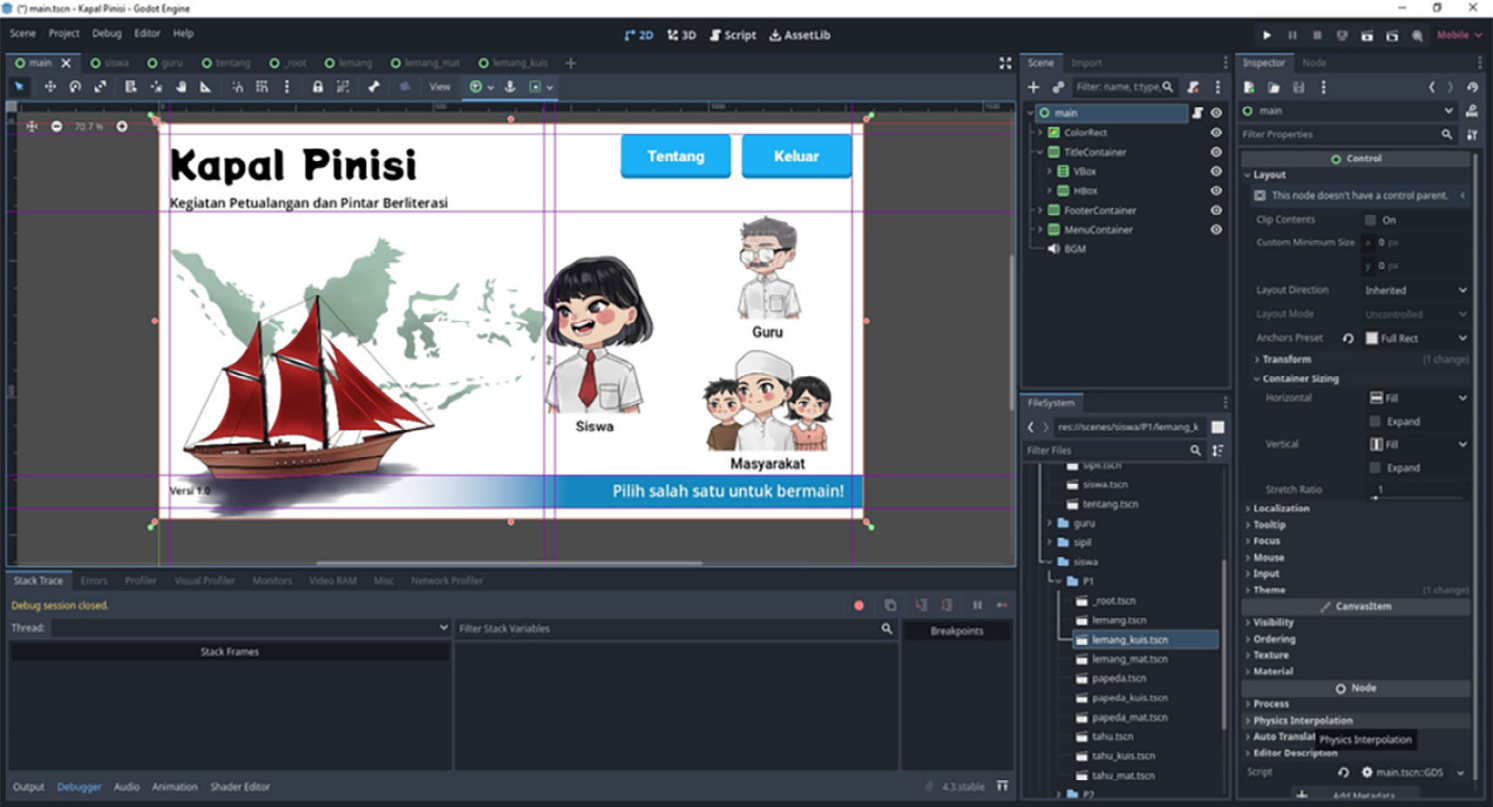
Main interface of the Kapal Pinisi digital learning application, illustrating the character selection menu and navigation layout designed to support multiliteracy and 6C competencies.

The main interface of the Kapal Pinisi Application, as illustrated in Figure 1, was designed with an intuitive layout to facilitate user navigation and engagement during literacy learning activities.

**Figure 2.**
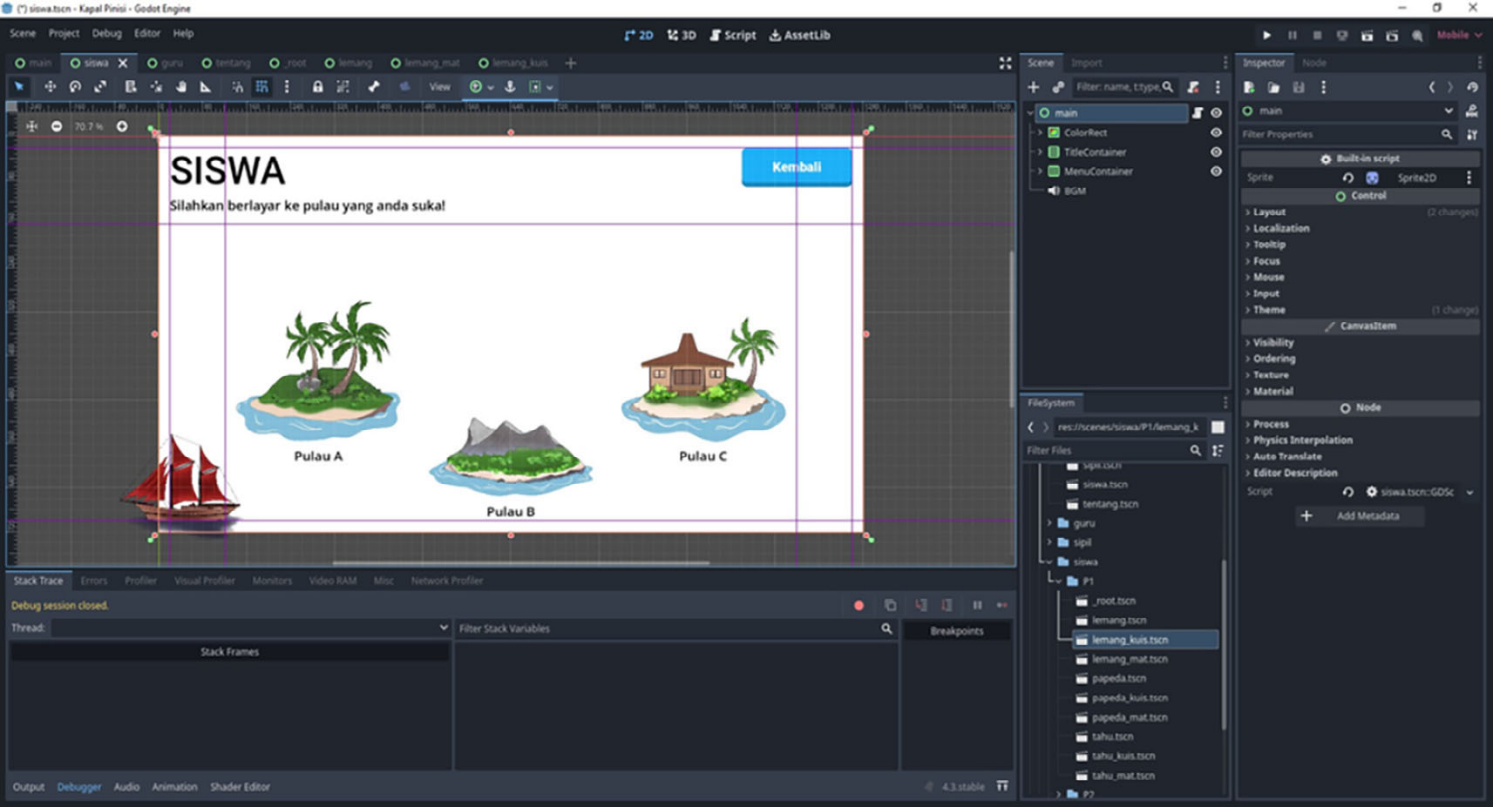
Second interface layout of the Kapal Pinisi digital learning application, illustrating the student module with selectable island destinations, as developed within the Godot Engine workspace.

The interface features include animated icons, navigation menus, and cultural story panels, as shown in Figure 2, which represent the main visual framework of the application.

**Figure 3.**
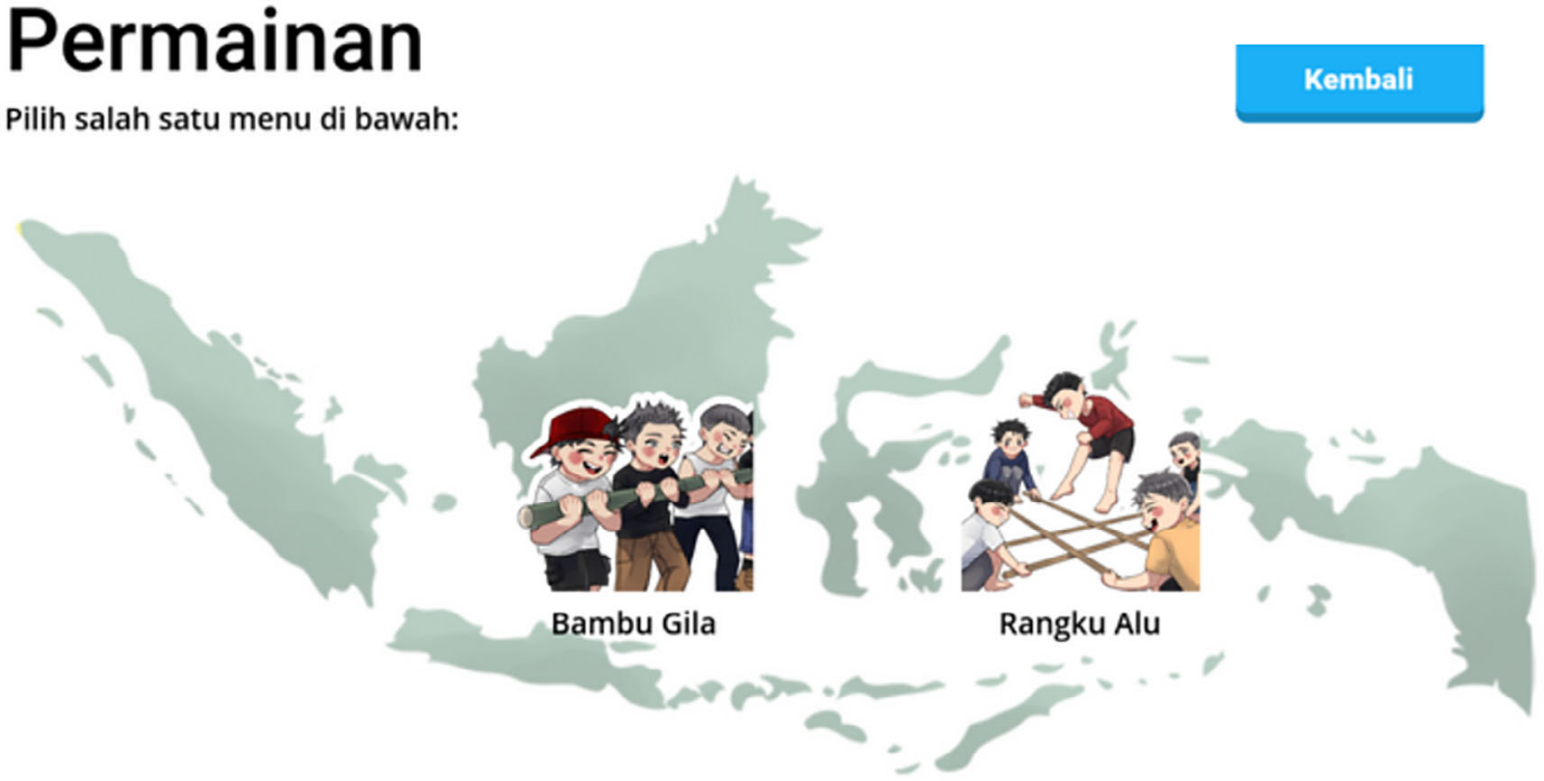
Third interface layout of the Kapal Pinisi digital learning application, illustrating the traditional games module featuring Bambu Gila and Rangku Alu.

The interactive simulation of traditional games, such as *congklak* and *spinning tops*, is depicted in Figure 3, demonstrating how cultural content is integrated into digital learning media.

**Figure 4.**
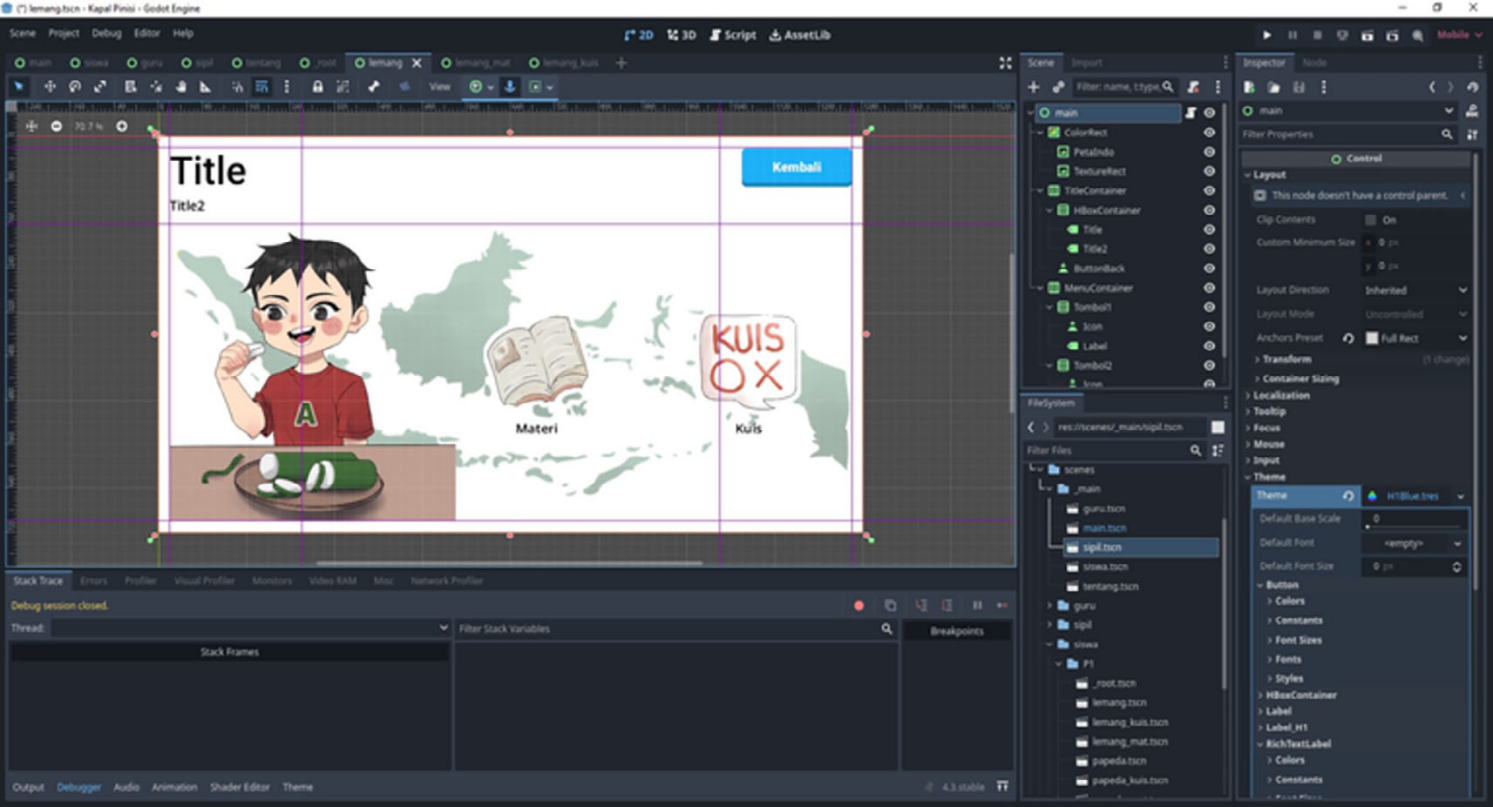
The instructional and quiz layout presenting evaluation items that measure basic literacy and cultural understanding.

The visual structure of the literacy quizzes and question formats can be seen in Figure 4, which illustrates how the evaluation component is embedded within the application design.

### Validity and reliability of instruments

The instruments used in this study consisted of pretests and posttests designed to measure students’ basic literacy and 6C competencies. Content validity was ensured through expert judgment by three specialists in elementary education and educational technology, who reviewed the test items for appropriateness and alignment with research objectives. Reliability testing was carried out using Cronbach’s Alpha, which yielded a coefficient of 0.86, indicating high internal consistency and confirming that the instruments were suitable for assessing the intended competencies.

### Ethical considerations

The research protocol was reviewed and approved by the Research Ethics Committee of Universitas Muhammadiyah Surakarta (Approval No: 5889/B.2/KEPK-FKUMS/IX/2025). Prior to data collection, informed consent was obtained from all participants involved in the study. For minor participants, written consent was obtained from parents or legal guardians, while verbal assent was obtained from the students themselves after a clear explanation of the study’s objectives, procedures, and their rights to withdraw at any time without penalty. The consent process ensured that participation was entirely voluntary, and all participants were informed that their academic performance would not be affected by their decision to participate or withdraw. Anonymity and confidentiality were strictly maintained throughout the research process. The official Ethical Clearance Letter confirming the approval has been deposited in Figshare and can be accessed via DOI: https://doi.org/10.6084/m9.figshare.30342175. ^23^

### Data analysis

The effectiveness of the **Kapal Pinisi** application was measured using pretest and posttest scores, which were analyzed through the N-Gain test to determine the degree of improvement in learning outcomes. Statistical analysis was performed using SPSS version 29 for Windows, with interpretation based on established N-Gain criteria from previous studies. ^24^ The detailed instruments used for the pretest and posttest have been archived in Figshare and are accessible via DOI: https://doi.org/10.6084/m9.figshare.30281275

The interpretation of the N-Gain results followed the standard criteria presented in Table 1, which classifies learning effectiveness into three categories: low, moderate, and high.

**Table 1.** Criteria for interpreting N-Gain test results, classifying learning effectiveness into low, moderate, and high levels.

Statistic (g)	Mean result	Interpretation	Effectiveness level
g < 0.3	Low	Ineffective	Not Effective
0.3 ≤ g ≤ 0.7	Moderate	Effective	Effective
g > 0.7	High	Highly Effective	Very Effective

N = Number of participants from the eight schools; Mean = Average improvement score; Std. Deviation = Variation of scores across students.

## Results and discussion

The development of the Kapal Pinisi application as a learning medium is motivated by the limited use of technology-based instructional tools, which has contributed to the low level of basic literacy, 6C competencies, and students’ understanding of local wisdom. Accordingly, the Kapal Pinisi application is designed to enhance students’ basic literacy and 6C skills through the integration of local wisdom into the learning process.

Findings indicated significant improvement in students’ literacy and 6C competencies. The average N-Gain score was 0.88, categorized as highly effective. Students showed improvement in reading comprehension, numeracy, creativity, collaboration, and character formation. The integration of local cultural elements increased engagement and contextual understanding.

### Analysis

In the analysis stage, a learning needs assessment was conducted to identify students’ characteristics and the relevant contextual factors. The findings indicated that students showed a strong preference for technology-based learning, particularly those designed in the form of games. This provides an opportunity to develop the Kapal Pinisi application as an interactive and engaging medium that fosters enjoyable learning experiences. Furthermore, integrating local wisdom is considered essential, as linking the learning content with local culture and traditions is expected to help students better understand and appreciate their sociocultural context.

In addition to technological preferences, the learning objectives were directed toward enhancing basic literacy and strengthening the 6C competencies, namely Critical Thinking, Creativity, Collaboration, Communication, Character, and Citizenship. Through the designed activities, students are expected not only to develop these competencies but also to understand and internalize the values of local wisdom. Thus, the analysis stage aims to design a learning experience that promotes both literacy and 21st-century skills while simultaneously reinforcing students’ cultural identity.

### Design

At the design stage, a detailed plan was formulated to develop the Kapal Pinisi application as a learning medium. The application was structured with an intuitive and engaging main menu, enabling students to easily navigate and access its features. The main menu includes several key elements, such as a clear and prominent title that captures students’ attention. Additionally, the application provides access to three virtual islands, each containing different aspects of local wisdom. These islands are designed to introduce cultural values and local traditions through interactive content that enhances students’ understanding. The application also features quizzes to assess students’ knowledge while motivating them to participate actively. To further increase visual appeal and student engagement, the application incorporates animated illustrations supporting the learning materials and background music that creates a positive learning atmosphere. The buttons are designed to be simple and user-friendly, ensuring smooth navigation without confusion.

In its design, particular emphasis was placed on strengthening students’ basic literacy and 6C competencies. Each interactive element is intended to foster critical thinking, collaboration, communication, and creativity. Through the strong integration of local wisdom, the Kapal Pinisi application is expected to provide an enjoyable and meaningful learning experience while simultaneously enhancing students’ literacy and 6C skills.

### Development

At the development stage, the design plan was implemented into a tangible product. The process began with the preparation of learning materials to support the Kapal Pinisi application. The research team collaborated with developers to produce high-quality content in the form of texts, images, and animations that reflect local wisdom. Each material was designed to be engaging and easily understood by students through simple language and appealing illustrations.

The next focus was on building the application itself. The software development team created an intuitive interface to ensure ease of use for young learners. Core features such as the three virtual islands, quizzes, and animations were optimized to function across multiple devices, including tablets and smartphones, thereby supporting technology-based learning. With this structured development, the application is expected to effectively enhance students’ basic literacy and 6C competencies while simultaneously introducing local wisdom in an interactive and enjoyable way.

### Implementation

At the implementation stage, the Kapal Pinisi application was tested on two different scales, namely small-scale and large-scale trials, to evaluate its effectiveness in enhancing students’ basic literacy and 6C competencies.

The summary of results from the small-scale trial is shown in Table 2, which provides descriptive statistics of students’ learning improvement after initial implementation.

**Table 2.** Results of the small-scale trial including mean and standard deviation values of students’ literacy improvement.

N	Mean	Std. deviation
50	0,48	0,31758

N = Number of participants in the small-scale trial; Mean = Average literacy improvement score; Std. Deviation = Variation of students’ literacy improvement scores.

The small-scale trial was conducted with a limited group of participants. Prior to using the application, students completed a pretest to assess their initial level of basic literacy and understanding of local wisdom. After using the application, they undertook a posttest to measure the learning gains. The instruments used in both the pretest and posttest, as well as the corresponding trial data, have been archived in Figshare and are accessible via DOI: https://doi.org/10.6084/m9.figshare.30278764. ^25^ The data from both tests were analyzed using the N-Gain test to determine the degree of improvement. The results of the small-scale trial revealed an N-Gain value of 0.48 with a standard deviation of 0.31758, which falls into the medium category and is considered effective. Further analysis showed that the most notable improvements occurred in students’ **basic literacy skills, particularly reading comprehension and numeracy**, as well as in selected dimensions of the **6C competencies, including collaboration and communication**. These findings indicate that even at the initial stage, the **Kapal Pinisi** application contributed positively not only to overall learning outcomes but also to specific domains of literacy and 21st-century skills, thereby providing a strong foundation for broader implementation.

The outcomes of the large-scale trial are detailed in Table 3, illustrating a substantial increase in students’ basic literacy and 6C competencies across multiple schools.

**Table 3.** Results of the large-scale trial across eight elementary schools, showing a high level of effectiveness in improving basic literacy and 6C competencies.

N	Mean	Std. deviation
143	0,88	0,16172

N = Total number of participants from eight elementary schools; Mean = Average literacy improvement score; Std. Deviation = Variation of literacy outcomes across students.

The large-scale trial was subsequently carried out with a greater number of students to obtain more representative results. The procedure followed the same steps, with pretests administered before the use of the application and posttests conducted after the learning process. The data were again analyzed using the N-Gain test. The dataset for the large-scale trial, along with supporting statistical analyses, is included in the same Figshare repository ( https://doi.org/10.6084/m9.figshare.30278764). ^26^ The findings showed an N-Gain value of 0.88 with a standard deviation of 0.16172, categorized as high and highly effective. In particular, students demonstrated substantial improvements in **basic literacy skills, especially reading comprehension, writing fluency, and numeracy**, along with significant gains in multiple **6C competencies, including critical thinking, creativity, collaboration, and character development**. These results demonstrate that the **Kapal Pinisi** application is not only beneficial but also highly successful in strengthening both literacy and a broad spectrum of 21st-century skills. By integrating elements of local wisdom into an interactive digital medium, the application enriched the learning experience, reinforced cultural understanding, and fostered essential competencies needed to meet the demands of modern education.

### Evaluation

The evaluation stage represents the final phase of the ADDIE development model, which serves to review all preceding stages, namely analysis, design, development, and implementation. At this stage, the Kapal Pinisi application was evaluated through students’ pretests and posttests to measure its effectiveness. The results indicated that the application succeeded in making the learning process more engaging and enjoyable, thereby facilitating better delivery of materials. Furthermore, students demonstrated improvements in basic literacy and 6C competencies integrated with local wisdom. This highlights that evaluation not only functions as the concluding step but also as a means of ensuring that learning objectives are achieved effectively through relevant media tailored to students’ needs.

This research produced the Kapal Pinisi application, a local wisdom–based digital learning medium designed to enhance elementary students’ basic literacy and 6C skills. The findings confirmed that technology-based learning can significantly improve literacy mastery and 21st-century competencies. Through the systematic ADDIE stages, the application successfully integrated elements of local wisdom in an engaging and interactive manner. These outcomes align with prior studies emphasizing the role of game-based media in increasing students’ engagement and motivation. ^27, 28^ Accordingly, the Kapal Pinisi application is considered highly effective as a learning medium that combines technological innovation with cultural values, contributing to the enhancement of students’ learning outcomes, particularly in basic literacy and 6C competencies.

## Conclusions

The findings of this study demonstrate that the *Kapal Pinisi Application* learning media, developed through the five stages of the ADDIE model, is both feasible and highly effective for classroom use. Its effectiveness is evident from the N-Gain analysis of students’ pretest and posttest scores, which yielded a value of 0.88, indicating a high level of improvement. This result confirms that the *Kapal Pinisi Application* is a very effective tool for enhancing students’ basic literacy and fostering 21st-century competencies, particularly the 6Cs, in elementary education. Nevertheless, this study has certain limitations, especially regarding the scope of the content, which is currently limited to the integration of local wisdom. Therefore, further research is recommended to expand the application’s content coverage, enabling it to serve as a more comprehensive medium that not only enriches students’ learning experiences but also strengthens character development through both local wisdom and broader scientific contexts. The Kapal Pinisi Application, integrating technology and local wisdom, proved highly effective in enhancing basic literacy and 6C competencies among elementary students. This innovation provides a validated model for culturally grounded, technology-enhanced learning that aligns with 21st-century education and sustainability goals.

## Data Availability

All data underlying the results reported in this article are freely available in open access repositories under a Creative Commons Attribution (CC-BY) license. The datasets include all numerical values behind means, standard deviations, and other statistical measures; data used to construct figures and tables; extracted points for analysis; and descriptive variables such as participants’ age, gender, and school characteristics.
1. Ethical approval document: Figshare. https://doi.org/10.6084/m9.figshare.30342175 ^23^
Data are available under terms of the https://creativecommons.org/licenses/by/4.0/ (CC-BY 4.0) 2. Pretest and posttest instruments: Figshare. https://doi.org/10.6084/m9.figshare.30281275 ^25^
Data are available under terms of the https://creativecommons.org/licenses/by/4.0/ (CC-BY 4.0) 3. Small-scale and large-scale trial data: Figshare. https://doi.org/10.6084/m9.figshare.30278764 ^26^
Data are available under terms of the https://creativecommons.org/licenses/by/4.0/ (CC-BY 4.0) All datasets are openly accessible without embargo or login requirements and can be reused under the CC-BY license for educational and non-commercial purposes. No data have been withheld due to ethical, privacy, or security restrictions.

## References

[1] Marmoah S Poerwanti E Suharno. : Literacy culture in elementary schools: planning, habituation, and evaluation. *Heliyon.* 2022;8(11):e11423. 35520627 10.1016/j.heliyon.2022.e09315 PMC9062677

[2] International Journal of Language Education: The correlation between textbook quality and basic literacy skills of lower elementary school students. *Int. J. Lang. Educ.* 2022;6(2):45–57.

[3] Damariswara R Laila A Mujiwati ES : Analysis of Needs for Developing KAPAL PINISI Application (Smart Literacy Adventure Activity) Based on Local Wisdom to Form 6C Skills of Indonesian Society. *Jurnal Pendidikan Dasar Nusantara.* 2025;10(2):248–260. 10.29407/jpdn.v10i2.24007

[4] Budiarto MK Asrowi G Karsidi R : E-Learning Platform for Enhancing 21st Century Skills for Vocational School Students: A Systematic Literature Review. *Electronic Journal of e-Learning.* 2024;22(5):76–90. 10.34190/ejel.22.5.3417 Academic Publishing

[5] Hafiz M Agustini K Suartama IK : Blended Learning and its Impact on 21st Century Student Learning. *Indonesian Journal of Innovation Studies.* 2025;26(3). 10.21070/ijins.v26i3.1449

[6] Yetti E Asmayawati Yufiarti : Pedagogical innovation and curricular adaptation in enhancing digital literacy: A local wisdom approach for sustainable development in Indonesia context. *J. Open Innov.: Technol. Mark. Complex.* 2024;10(1):100233. 10.1016/j.joitmc.2024.100233

[7] Drake SM : Why 21st century competencies can’t be an afterthought. *Frontiers in Education.* 2020;5:122. 10.3389/feduc.2020.00122

[8] Sakti SA Endraswara S Rohman A : Revitalizing local wisdom within character education through ethnopedagogy approach: A case study on a preschool in Yogyakarta. *Heliyon.* 2024;10:e31370. 10.1016/j.heliyon.2024.e31370 38803959 PMC11129093

[9] The New London Group: A pedagogy of multiliteracies: Designing social futures. *Harv. Educ. Rev.* 1996;66(1):60–93. 10.17763/haer.66.1.17370n67v22j160u

[10] Walsh M : Multiliteracies: New literacies and digital epistemologies. *Aust. J. Lang. Lit.* 2010;33(1):211–239. 10.1007/BF03651836

[11] Alismail HA McGuire P : The 21st century skills and their impact on students’ learning. *J. Educ. Pract.* 2015;6(6):150–154.

[12] McGuire: OECD, 2021. 2015.

[13] Basri H Rahayu S Lestari D : Local wisdom as a foundation for character education in the digital era. *Journal of Ethnopedagogy.* 2022;4(1):55–66.

[14] Laila A Asri CB Syamsi K : Textbooks based on local wisdom to improve reading and writing skills of elementary school students. *International Journal of Evaluation and Research in Education.* 2021;10(1):89–96. 10.11591/ijere.v10i1.20755

[15] Suwandi S Hidayat R Nurjanah I : Civic literacy through cultural values: strengthening character education. *Civic Education Journal.* 2020;10(3):210–222.

[16] Ng W : Can we teach digital natives digital literacy? *Comput. Educ.* 2012;59(3):1065–1078. 10.1016/j.compedu.2012.04.016

[17] Hatlevik OE Guðmundsdóttir GB Loi M : Digital diversity among upper secondary students: A multilevel analysis. *Comput. Educ.* 2018;122:1–14.

[18] Siddiq F Scherer R : The relation between students’ digital literacy and learning outcomes: a meta-analysis. *Educ. Res. Rev.* 2019;28:100–118.

[19] Hoskins B Janmaat JG Villalba C : Civic engagement and education for social cohesion. *Eur. J. Educ.* 2022;57(1):23–39.

[20] UNESCO: *Global Education Monitoring Report 2019: Building bridges for social solidarity.* Paris: UNESCO Publishing;2019.

[21] Fitriani L Handayani R Suryono B : Implementation of ADDIE model in developing learning media. *Jurnal Teknologi dan Pembelajaran.* 2024;9(2):101–112.

[22] Nengseh N Damayanti R : N-Gain analysis for evaluating learning effectiveness. *J. Educ. Meas.* 2022;8(1):34–40.

[23] Gunawan Laila A : Ethical clearance letter for the research “Development of KAPAL PINISI (Smart Literacy Adventure Activities) Application Based on Local Wisdom to Foster 6C Skills” [Ethical approval document]. *Figshare.* 2025. 10.6084/m9.figshare.30342175

[24] Abidin Y Mulyati T Yunansah H : Game-based learning and its impact on student motivation. *Journal of Educational Research and Evaluation.* 2022;11(3):233–245.

[25] Gunawan Laila A : Pretest and posttest instruments for the study “Development of the Kapal Pinisi Application (Smart Literacy Adventure Activities) Based on Local Wisdom to Foster Basic Lietracy and 6C Skills.” *Figshare.* 2025. 10.6084/m9.figshare.30281275

[26] Gunawan Laila A : Small-scale and large-scale trial data for the study “Development of the Kapal Pinisi Application (Smart Literacy Adventure Activities) Based on Local Wisdom to Foster Basic Lietracy and 6C Skills.” *Figshare.* 2025. 10.6084/m9.figshare.30278764

[27] Rachmawati D Hartati T Wibowo A : The role of learning media in increasing students’ engagement. *Jurnal Pendidikan Dasar Indonesia.* 2023;8(1):12–25.

[28] Krisbiantoro A Santosa H Putri D : Technology integration in elementary education: challenges and opportunities. *Jurnal Teknologi Pendidikan.* 2021;23(2):99–110.

