# Supplementary material for: Transforming Basic Literacy Instruction through the Kapal Pinisi Application: Integrating Local Wisdom to Foster Basic Literacy and 6C Competencies in Elementary School Students
Source: F1000Res. 2026 Mar 4;15:355. [Version 1] doi: 10.12688/f1000research.172137.1 (PMC13182781; doi:10.12688/f1000research.172137.1)
Supplement: Peer review history

# Reviewer report: Reviewer response for version 1

**DOI:** 10.5256/f1000research.189835.r465649

**Published:** 2026-03-31

**Authors:** Nurul Mahruzah Yulia

**Affiliations:** 1 Universitas Nahdlatul Ulama Sunan Giri Bojonegoro, Bojonegoro, East Java, Indonesia

The article *“Transforming Basic Literacy Instruction through the Kapal Pinisi Application”* presents a relevant and innovative effort to integrate local wisdom with digital learning in order to improve elementary students’ basic literacy and 6C competencies. The use of a Research and Development (R&D) approach through the ADDIE model is appropriate for developing instructional media, and the stages are described systematically, including analysis, design, development, implementation, and evaluation.

  Another major issue concerns the measurement of the 6C competencies. Although the study claims to improve Critical Thinking, Creativity, Communication, Collaboration, Citizenship, and Character, it does not clearly explain how each of these competencies is defined, measured, or assessed. There is no detailed description of indicators, scoring procedures, or types of assessment used, which raises concerns about construct validity. Given that 6C competencies are complex and multidimensional, their assessment requires clear operational definitions and appropriate measurement tools, which are not sufficiently presented in the Methods section

Is the work clearly and accurately presented and does it cite the current literature?

Yes

If applicable, is the statistical analysis and its interpretation appropriate?

Partly

Are all the source data underlying the results available to ensure full reproducibility?

Yes

Is the study design appropriate and is the work technically sound?

Yes

Are the conclusions drawn adequately supported by the results?

Yes

Are sufficient details of methods and analysis provided to allow replication by others?

Partly

Reviewer Expertise:

Digital, Literacy, Elementary School.

I confirm that I have read this submission and believe that I have an appropriate level of expertise to confirm that it is of an acceptable scientific standard.

# Reviewer report: Reviewer response for version 1

**DOI:** 10.5256/f1000research.189835.r465648

**Published:** 2026-03-30

**Authors:** Rasmitadila Rasmitadila

**Affiliations:** 1 Universitas Djuanda, Bogor, Indonesia

Overall, this article has demonstrated good innovation in integrating technology and local wisdom to improve 6C literacy and competency, but still needs improvement in the methodological aspects so that the results are more scientifically robust; researchers are advised to add a control group to strengthen the effectiveness claim, explain in more detail the operationalization and measurement instruments for each 6C component, clarify the implementation procedure (duration, learning stages, and teacher roles), and complement the data analysis with inferential statistical tests (not only N-Gain) so that the conclusions drawn do not seem too general and are more academically accountable.

Is the work clearly and accurately presented and does it cite the current literature?

Yes

If applicable, is the statistical analysis and its interpretation appropriate?

Partly

Are all the source data underlying the results available to ensure full reproducibility?

Yes

Is the study design appropriate and is the work technically sound?

Yes

Are the conclusions drawn adequately supported by the results?

Yes

Are sufficient details of methods and analysis provided to allow replication by others?

Partly

Reviewer Expertise:

Inclusive education in elementary school; teacher development

I confirm that I have read this submission and believe that I have an appropriate level of expertise to confirm that it is of an acceptable scientific standard.

# Reviewer report: Reviewer response for version 1

**DOI:** 10.5256/f1000research.189835.r465644

**Published:** 2026-03-18

**Authors:** Bramianto Setiawan

**Affiliations:** 1 Universitas Pelita Bangsa, Cikarang, West Java, Indonesia

**Full Review Report**

 This manuscript addresses an educationally relevant topic by presenting the development and evaluation of a digital learning medium (the Kapal Pinisi Application) to improve elementary school students' basic literacy skills and 6C competencies through the integration of local wisdom. This topic is timely and potentially valuable, particularly in the Indonesian context, where culturally responsive and technology-supported literacy interventions are urgently needed. The manuscript also has a practical orientation and benefits from the availability of open data.

 However, despite the topical relevance, the current version of the manuscript has substantial methodological and analytical weaknesses that prevent its main claims from being adequately supported. Currently, the manuscript reads more like a development report than a scientifically sound effectiveness study. The main issue is that the authors make strong claims regarding effectiveness, improvement of all 6C competencies, and broader educational relevance, while the design, measurement, and analysis do not provide sufficient evidence to justify these conclusions.

 The manuscript therefore requires **major revision**.

**Summary of the article**

 The paper reports a Research and Development study using the ADDIE model to develop the *Kapal Pinisi Application*, a local wisdom–based digital learning application for elementary school students. The intervention was tested through small-scale and large-scale implementation stages using pretest–posttest assessment, and effectiveness was evaluated primarily through N-Gain analysis. The authors report an average N-Gain of 0.88 in the large-scale trial and conclude that the application is highly effective in improving basic literacy and 6C competencies.

**Major concerns**

**1. The study design is not sufficiently rigorous to support the strength of the effectiveness claims.**

 The use of the ADDIE model is appropriate for instructional product development. However, the manuscript moves beyond development and makes strong effectiveness claims without employing a design capable of supporting such claims convincingly. The evaluation relies on pretest–posttest comparisons and N-Gain analysis only, with no control or comparison group. As a result, the study cannot isolate the effect of the application from alternative explanations such as teacher influence, testing effects, maturation, novelty effects, or contextual differences across schools.

 This is not a minor limitation. It directly affects the validity of the paper’s main conclusion. The authors should not describe the application as “proved highly effective” or present it as a validated model in a broad sense when the underlying design does not support causal inference. At minimum, the conclusions must be substantially toned down. Ideally, the authors should reframe the paper as a developmental study with preliminary evidence of promise, not as a strong effectiveness study.

**2. The manuscript does not adequately operationalize or measure the 6C competencies.**

 A major conceptual and methodological weakness is the lack of clarity regarding how the six competencies were defined, assessed, and analyzed. The article repeatedly states that the application improves Critical Thinking, Creativity, Communication, Collaboration, Citizenship, and Character, yet the manuscript does not provide a sufficiently explicit measurement framework for these constructs.

 It remains unclear:

- what indicators were used for each competency,
- whether all six competencies were measured directly,
- whether they were measured through tests, observations, ratings, or performance tasks,
- how scores were computed,
- and how validity was established for this multidimensional measurement claim.

This omission is serious because the 6C framework is central to the article’s novelty and contribution. Without a transparent operational definition and corresponding evidence, the claims regarding improvement in 6C competencies are currently under-substantiated. The manuscript needs a dedicated table or subsection specifying each competency, its indicators, instrument source or development basis, number of items, scoring method, and evidence of validity.

**3. The Methods section does not provide enough detail for replication.**

 The manuscript provides a general overview of the ADDIE stages and sample distribution but lacks important procedural details. Replication would be impossible based on the current description. For example, the authors do not clearly report:

- the duration of the intervention,
- the number of instructional sessions,
- the length of each session,
- the instructional sequence,
- the extent of teacher guidance,
- whether implementation was standardized across schools,
- and how implementation fidelity was ensured.

Similarly, although the pre- and post-test instruments are mentioned and archived externally, the manuscript does not fully explain their composition. Readers need sufficient information in the manuscript to understand what was measured and how. This section should be substantially expanded. Currently, this study is not reported with the expected level of methodological transparency.

**4. The statistical analysis is too limited for the claims being made.**

 The use of N-Gain as a descriptive indicator of learning improvement is acceptable in educational research. However, N-Gain alone is insufficient to support the broader claims made in the manuscript. The paper reports mean N-Gain values ​​and interprets them as evidence of high effectiveness but does not provide inferential statistical testing to confirm whether the observed pre-test and post-test differences are statistically significant. The paper also does not provide separate analyses for literacy outcomes and each of the six claimed C-dimensions.

 This is particularly problematic because the paper repeatedly claims improvements across multiple constructs. If the authors want to argue that the intervention improved literacy and multiple competencies, the results section should present fragmented evidence rather than broad narrative statements. Currently, the statistical reporting is too thin compared to the scope of the conclusions.

 The authors should strengthen this section by adding appropriate inferential analysis, clarifying the level of N-Gain calculation, reporting descriptive statistics for pre-test and post-test scores directly, and presenting results separately by domain where relevant. If such analysis is not available, the claim needs to be narrowed accordingly.

**5. There is inconsistency in sample reporting.**

 The manuscript states that 200 students participated, consisting of 50 in the small-scale trial and 150 in the large-scale trial. However, the large-scale results table reports N = 143. This discrepancy should be explained. Missing data, attrition, exclusion criteria, or incomplete cases may be valid but must be explicitly reported.

 Authors should provide a clear explanation of the participant flow, including the number recruited the number who completed each phase, the number included in the analysis, and any exclusions.

**6. Conclusions substantially exceed the available evidence.**

 The current conclusions are written in a way that goes beyond what research can support. The manuscript states that the app is highly effective, feasible, validated, and aligned with sustainability goals, and shows potential for broader strategic educational use. These claims are premature given limitations in design, measurement, and analysis. A more scientifically sound conclusion would be that the app showed promising initial results during development trials and is worthy of further testing under more rigorous evaluation conditions.

Is the work clearly and accurately presented and does it cite the current literature?

Yes

If applicable, is the statistical analysis and its interpretation appropriate?

Partly

Are all the source data underlying the results available to ensure full reproducibility?

Yes

Is the study design appropriate and is the work technically sound?

Partly

Are the conclusions drawn adequately supported by the results?

Partly

Are sufficient details of methods and analysis provided to allow replication by others?

Partly

Reviewer Expertise:

Elementary education, science education in primary schools, STEM/STEAM education, educational technology and learning media development, and sustainable development (SDGs) integration in primary education.

I confirm that I have read this submission and believe that I have an appropriate level of expertise to confirm that it is of an acceptable scientific standard, however I have significant reservations, as outlined above.
